# Characterization of differential expression and leader intron function of *Arabidopsis atTOC159* homologous genes by transgenic plants

**DOI:** 10.1186/1999-3110-54-40

**Published:** 2013-09-25

**Authors:** Yu-Shan Liu, Chih-Wen Sun

**Affiliations:** Department of Life Science, National Taiwan Normal University, Taipei, 116 Taiwan

**Keywords:** *Arabidopsis thaliana*, *atTOC159* homologous genes, Transgenic plant, Promoter activity, Reporter gene, Leader intron

## Abstract

**Background:**

Accurate import of thousands of nuclear-encoded proteins is an important step in plastid biogenesis. However, the import machinery of cytosolic precursor proteins to plastids relies on the Toc and Tic (translocons on the outer envelope and inner envelope membrane of chloroplasts) complexes. Toc159 protein was identified in pea (*Pisum sativum*) as a major receptor for the precursor proteins. In *Arabidopsis thaliana*, four psToc159 homologs are identified, termed atToc159, atToc132, atToc120 and atToc90. The expression of these protein-encoding genes has to be properly regulated, because their gene products must be correctly integrated to appropriate apparatus to perform their functions.

**Results:**

In order to elucidate the regulatory mechanisms of *atTOC159* homologous gene expression, transgenes containing various lengths of the upstream regulatory sequences of *atTOC159*/*atTOC132*/*atTOC120*/*atTOC90* and *GUS* coding sequence were transferred to wild type *Arabidopsis*. In accordance with the analysis of GUS activity in these transgenic plants at various developmental stages, these homologous genes had distinct expression patterns. *AtTOC159* and *atTOC90* are preferentially expressed in above-ground tissues, such as cotyledons and leaves. In mature roots, *atTOC159* and *atTOC132* are expressed at higher levels, while *atTOC120* and *atTOC90* are expressed at the basal level. All four genes have increased expression level during flower and fruit development, particularly a remarkably high expression level of *atTOC159* in later stage of fruit development. Furthermore, leader intron in the 5′ UTR induces the expression level of *atTOC159* members in a tissue-specific manner. This is able to up-regulate the *atTOC120* expression in roots/leaves/flowers, and the *atTOC90* expression in cotyledons/leaves/anthers.

**Conclusions:**

The differential expression of *atTOC159* gene members is essential during plastid development, because proper atToc159 isoforms are required to import distinct proteins to the plastids of different tissues.

**Electronic supplementary material:**

The online version of this article (doi:10.1186/1999-3110-54-40) contains supplementary material, which is available to authorized users.

## Background

From an engulfed cyanobacterial ancestor, plastid is nowadays converted into three major groups in the plant cell, according to its function and location. Chloroplasts ubiquitously exist in the cells of green tissues, and are responsible for photosynthesis, the synthesis of amino acids and lipids, the synthesis of phytohormones, and the storage of starch and oil compounds. Chromoplasts appear in the cells of leaves, flowers and fruits, and are predominantly responsible for the synthesis of pigments. Amyloplasts majorly exist in the root cells and minor in the stem cells, functioning as the storage of starch and the gravity sensor. However, more than 90% of the plastid genes have since been transferred to the host nucleus during evolution (Martin et al., 2002). In consequence, these nuclear-encoded plastid-localized proteins must be correctly imported to the plastids during the processes of plastid biogenesis to guarantee proper plastid functions.

Except for a few outer membrane proteins, most of the nuclear-encoded plastid proteins are imported into plastids via a set of translocon components located at the outer/inner envelope membrane of chloroplasts (Toc and Tic; Inaba and Schnell, 2008). Toc34 and Toc159 are Toc core-complex components, and also identified as GTPases. They are in charge of recognizing preprotein, and thus are regarded as receptors for the preprotein (Jarvis, 2008). In *Arabidopsis*, Toc34 and Toc159 are encoded by multiple genes. *AtTOC33* (At1g02880) and *atTOC34* (At5g05000) are homologous to pea *psTOC34* (Jarvis et al., 1998), whereas *atTOC159* (At4g02510), *atTOC132* (At2g16640), *atTOC120* (At3g16620) and *atTOC90* (At5g20300) are homologous to *psTOC159* (Bauer et al., 2000). Genetic and biochemical analyses revealed that different receptor isoforms encoded by these gene families had distinct functions. AtToc33 and atToc159/atToc90 are probably the receptors specific for photosynthesis-related proteins (Jarvis et al., 1998;Bauer et al., 2000;Kubis et al., 2003;Hiltbrunner et al., 2004). However, atToc34 and atToc132/atToc120 are prone to recognize non-photosynthetic proteins and import them into plastids (Ivanova et al., 2004;Kubis et al., 2004).

Since atToc159 protein isoforms might recognize and allow to import diverse plastid proteins, *atTOC159* homologous genes should be independently regulated during the process of vegetative and reproductive development. In fact, the regulation of *atTOC159* gene members in several organs had been reported. Bauer et al. (2000) showed that the expression of the mRNA level of *atTOC159* was five to ten times more than that of *atTOC132* and *atTOC120* in both 6-day-old etiolated and green seedling, indicating that *atTOC159* is predominantly expressed in young seedling. Furthermore, Kubis et al. (2004) showed that *atTOC159* was strongly expressed in 10-day-old leaves, *atTOC90* was expressed at a uniformly moderate level in leaves/inflorescence/roots, whereas *atTOC132* and *atTOC120* were expressed at a uniformly low level but relatively prominent in inflorescence and roots. The expression level of *atTOC90* in 28-day-old leaves, inflorescence and roots were approximately 3 times, 1.3 times and 5 times, respectively, higher than that of *atTOC159*. By contrast, the Gene Chronologer tool in Genevestigator revealed that *atTOC159* had 4 to 5 times higher expression than *atTOC90* in mature leaves and inflorescence (Zimmermann et al., 2004). Additional analyses are clearly needed to elucidate these conflicting results.

In this study, the differential expression of *atTOC159* homologous genes regulated by their upstream sequence in the vegetative and reproductive tissues was analyzed. We first created stable transgenic plants expressing the *GUS* reporter gene driven by the upstream sequences of *atTOC159* family members and determined the GUS activity of these transgenic plants. Our results suggest that *atTOC159* and *atTOC90* are preferentially expressed in 1-and 2-week-old leaves, whereas *atTOC159* and *atTOC132* have relative higher expression levels in 21-day-old roots. All *atTOC159* homologous genes have increased expression level in flowers and siliques, especially a remarkably higher expression level of *atTOC159* in later stage of fruit development. The intron effect on the transcription activity was also examined by analyzing GUS activity in the stable transgenic plants. Our results show that the expression yield of *atTOC120* is dramatically up-regulated in root tip and flowers by its leader intron sequence (the intron nearest to the transcription start site), while that of *atTOC90* is significantly up-regulated in cotyledons and anthers.

## Methods

### Plant material and growth conditions

*Arabidopsis* seeds from wild type (ecotype Columbia) and transgenic plants were surface sterilized with 25% (v/v) commercial bleach. For preparation of rosette leaves, seeds were germinated on 1 × MS (Murashige and Skoog 1962) agar medium with Gamborg’s vitamins and 2% (w/v) sucrose. For preparation of flowers and siliques, seeds were directly sowed on soil. For preparation of roots, seeds were germinated and grown in distilled water for 5 days and then transferred to nutrient solution for 16 days by means of a hydroponic system (Araponics). The nutrient solution was renewed every three day and the recipe was based on Fan et al. (2009). Plants were grown at 22°C under a 16-h light/8-h dark cycle in white light (85 μmol m^-2^ sec^-1^) for various numbers of days.

### Plasmid construction and plant transformation

**Figure 1:**
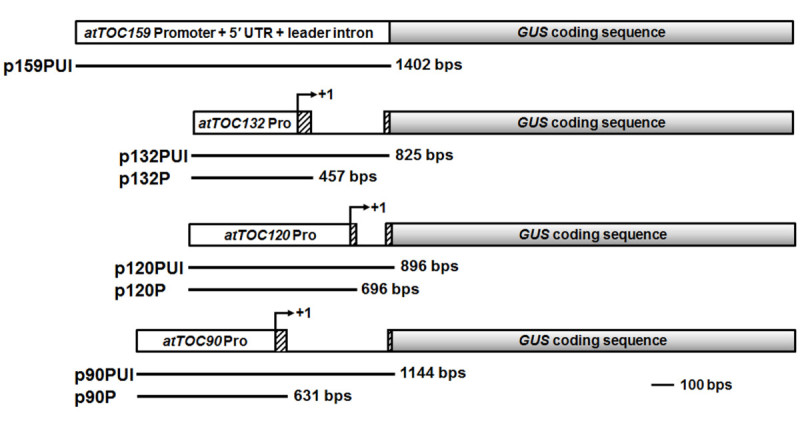
**Constructs containing the upstream sequences of** ***atTOC159*** **,** ***atTOC132*** **,** ***atTOC120*** **and** ***atTOC90*** **genes.** The p132PUI upstream sequence includes the promoter (−383 to −1), 5′ UTR (+1 to +74 and +428 to +444), and leader intron (+75 to +427) sequences of *atTOC132* gene. The p120PUI upstream sequence has the promoter (−673 to −1), 5′ UTR (+1 to +23 and +198 to +223), and leader intron (+24 to +197) sequences of *atTOC120* gene. The p90PUI upstream sequence has the promoter (−575 to −1), 5′ UTR (+1 to +56 and +563 to +570), and leader intron (+57 to +562) sequences of *atTOC90* gene. The transcription start site of *atTOC159* gene is undetermined, therefore the precise position of 5′ UTR and intron is unknown. The p132P, p120P and p90P constructs consist of the promoter and 5′ UTR sequences prior to the leader intron of *atTOC132*, *atTOC120* and *atTOC90*, respectively. Open box, promoter sequence. Hatched box, 5′ UTR sequence. Bronzed box, *GUS* coding sequence. Bold underline between the boxes, intron sequence. +1, transcription start site.

The upstream sequences of *atTOC159*, *atTOC132*, *atTOC120* and *atTOC90* genes were amplified by PCR and ligated into β-glucuronidase (GUS)-containing binary vector pCAMBIA1391Z (GenBank accession number AF234312). The resulting plasmids were transformed into wild type plants using the floral dipping method (Clough and Bent, 1998), mediated by *Agrobacterium* strain GV3101. The transformants were selected on agar plates containing 30 μg/mL hygromycin, and verified by PCR using construct-specific primers. These transformants were named 159PUI, 132PUI/132P, 120PUI/120P and 90PUI/90P, representing different lengths of upstream sequences, respectively (Figure 1). The 1391Z plant was transgenic plant containing pCAMBIA1391Z plasmid, and therefore served as negative control. The transgenic plants were selected for more than two generations and T_3_ homozygous transgenic plants were used for this study. The primer sequences specific for *atTOC159*, *atTOC132*, *atTOC120* and *atTOC90* upstream sequences are summarized in Table 1.

**Table 1:** **Sequences of gene-specific primers used in this study**

Gene	Forward primers (5′ to 3′)^a^	Reverse primers (5′ to 3′)^a^
*159PUI*	GCCAAGCTT GGATTTGTGTTATGTTTCTCGC	CGGGGATCC CCGCTTTGCTACTGAGACTC
*132PUI*	GCCAAGCTT TAGCTGCACCAGCTTATTGAG	CGGGGATCC TCTAGATCACCACCGCTACG
*132P*	GCCAAGCTT TAGCTGCACCAGCTTATTGAG	CGGGGATCC CAGAAGTTAGAGATAGAGAGAG
*120PUI*	GCCAAGCTT AGTGTGGTGTTGTTTAAGTGTG	CGGGGATCC CTAGGATCACCCAAAATCACG
*120P*	GCCAAGCTT AGTGTGGTGTTGTTTAAGTGTG	CGGGGATCC CTGGTTAGAGAAGGCAAAAGTC
*90PUI*	GCCAAGCTT AGACGAAGATGTCGTCATTGG	CGGGGATCC AACTATCTGCCCAACAGCAAG
*90P*	GCCAAGCTT AGACGAAGATGTCGTCATTGG	CGGGGATCC TTGTGTTGGCGAGAGAAAGAG

^a^The underlined sequences are additional restriction sites. *Bam* HI, GGATCC; *Hind* III, AAGCTT.

### GUS activity assays

The fluorometric quantification and histochemical localization of GUS enzyme activity were performed as described by Jefferson (1987). For fluorometric quantification, cell lysate was assayed for GUS activity with fluorometer (SpectraMax Gemini XPS, Molecular Devices). For histochemical staining, the plant tissues were incubated in GUS buffer for 1.5 to 24 hours at 37°C, and stored in fix solution (0.1 M sodium phosphate, pH 7.2, 0.1% formaldehyde, 0.1% triton X-100, 0.1% β-mercaptoethanol).

## Results

### Preferential expression of *atTOC159* and *atTOC90* in leaves

To determine whether the expression of *atTOC159* homologous genes was influenced by tissue-specific signals, promoter-GUS fusions were constructed for *atToc159* family members and analyzed in transgenic *Arabidopsis* plants. Approximately 1-kb upstream sequences of *atTOC159* homologs were individually placed into a binary vector pCAMBIA1391Z containing a GUS coding sequence as a reporter. The resulting constructs p159PUI, p132PUI, p120PUI and p90PUI contained a 1.4-kb, 0.82-kb, 0.89-kb and 1.14-kb upstream sequences of *atTOC159*, *atTOC132*, *atTOC120* and *atTOC90* genes, respectively (Figure 1). These plasmids were further transformed into wild type *Arabidopsis* by the floral dipping method. Twenty one, nine, nine and fourteen independent transgenic lines containing p159PUI, p132PUI, p120PUI and p90PUI, respectively, were obtained. These transgenic plants were named 159PUI-1 ~ 21, 132PUI-1 ~ 9, 120PUI-1 ~ 9, and 90PUI-1 ~ 14. Three homozygous T_3_ transformants representing each construct were used for further GUS analyses.

**Figure 2:**
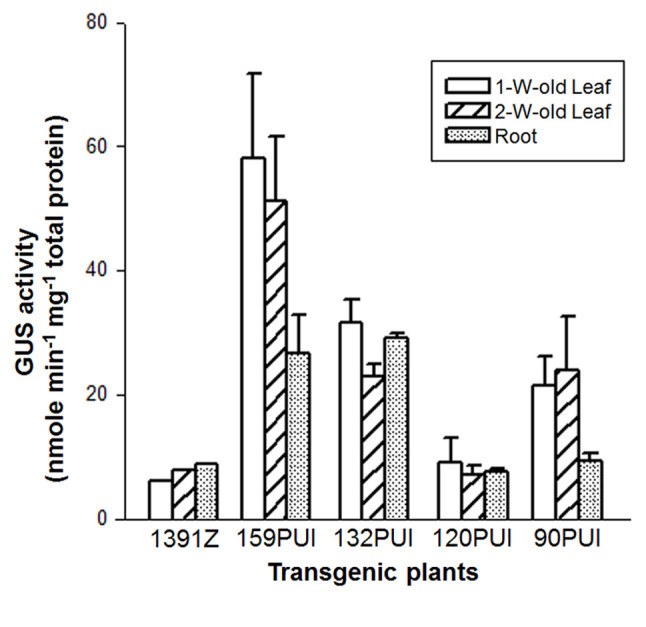
**Expression levels of** ***atTOC159*** **homologous genes in vegetative tissues.** The GUS activity of leaves and roots in transgenic plants 159PUI, 132PUI, 120PUI and 90PUI were determined. 1391Z plant is a negative control. Data are means ± SD of three repeat experiments from three individual transgenic lines (159PUI-1, 159PUI-3, 159PUI-8, 132PUI-6, 132PUI-7, 132PUI-8, 120PUI-1, 120PUI-3, 120PUI-9, 90PUI-1, 90PUI-3 and 90PUI-6).

The GUS activity in 1-to 2-week-old leaves of various transgenic plants was compared first. Among these transgenic plants, the GUS activity of 159PUI and 120 PUI transformants had the highest and lowest expression levels in 1-week-old leaves, respectively (Figure 2). Their GUS expression levels in 2-week-old leaves were not different from those in 1-week-old leaves. Both 132PUI and 90PUI plants had a moderate expression level in 1-week-old leaves. However, the expression was reduced to 75% of that for 132PUI plants in 2-week-old leaves, but remained steady for 90PUI plants. These results suggest that *atTOC159*, *atTOC120* and *atTOC90* are expressed at a constant level through the developmental stages of vegetative growth. In contrast, *atTOC132* expression was related to plant age, with highest promoter activity in the young leaves and a settled yield in the mature leaves. From above, it is obviously that *atTOC159* expression is predominantly and constantly expressed whereas *atTOC132* is modulated in an age-dependent manner in leaves.

Next, the expression differences of *atTOC159* homologous genes in 3-week-old roots were determined. The expression levels of *atTOC159* and *atTOC132* were similar and both were approximately three times higher than those of *atTOC120* and *atTOC90* (Figure 2). This reveals that *atTOC159* and *atTOC132* play a more significant role than others in roots. In fact, *atTOC120* and *atTOC90* seemed expressing at a basal level when compared to control construct p1391Z. This implies that *atTOC120* and *atTOC90* might not important for the root function. However, *atTOC90* expression in both 1-week-old and 2-week-old leaves was about 2.5 times higher than in roots, suggesting that *atTOC90* is preferentially expressed in leaves. Similar to the expression pattern of *atTOC90*, *atTOC159* expression in leaves was approximately 2 times higher than in roots, indicating that *atTOC159* is also preferentially expressed in leaves. Altogether, among the members of *atTOC159* gene family in *Arabidopsis*, the expression level of *atTOC159* is the highest in all vegetative tissues and *atTOC159* preferentially expressed in leaves. The *atTOC132* is moderately expressed in all tissues and is regulated in an age-specific manner in leaves. The *atTOC120* is poorly expressed in all tissues. Finally, *atTOC90* is modestly and preferentially expressed in leaves, but barely expressed in roots.

### Differential expression of *atTOC159* homologous genes in reproductive tissues

**Figure 3:**
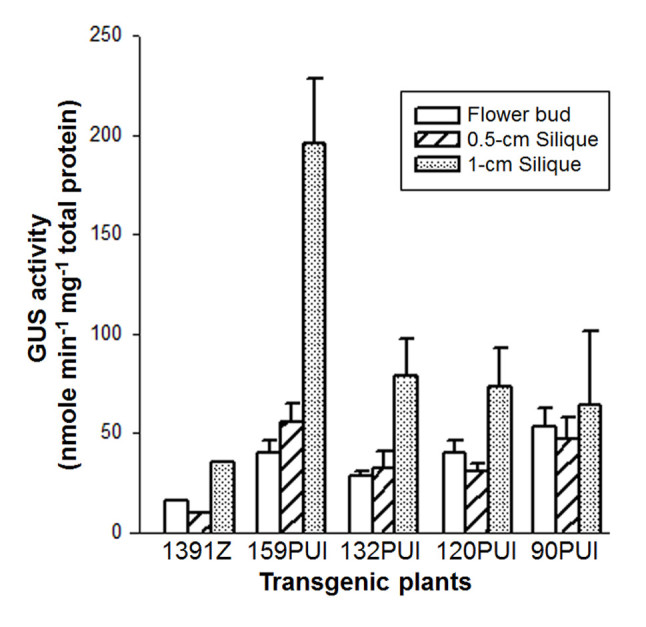
**Expression of** ***atTOC159*** **homologous genes in reproductive tissues.** The GUS activity of flower bud, 0.5-cm silique and 1-cm silique in transgenic plants 159PUI, 132PUI, 120PUI and 90PUI were determined. 1391Z plant is a negative control. Data are means ± SD of three repeat experiments from three individual transgenic lines (159PUI-1, 159PUI-3, 159PUI-8, 132PUI-6, 132PUI-7, 132PUI-8, 120PUI-1, 120PUI-3, 120PUI-9, 90PUI-1, 90PUI-3 and 90PUI-6).

In order to understand if the expression of *atTOC159* homologous genes was modulated by developmental signals in reproductive tissues, GUS activity of flower buds and different stages of siliques collected from various transgenic plants was determined. In flower buds and 0.5-cm siliques, the GUS activity of 159PUI, 132PUI, 120PUI and 90PUI was alike and about 2 to 3 times higher than that of control plant 1391Z (Figure 3). In 1.0-cm siliques, the GUS activity of 132PUI, 120PUI and 90PUI remained about 2 times higher than that of 1391Z, but the GUS activity of 159PUI was 4.6 times higher than that of 1391Z (Figure 3). These data indicate that *atTOC159*, *atTOC132*, *atTOC120* and *atTOC90* all participate in the regulation of the flower and fruit development. In addition, *atTOC159* appears to have a greater role during late fruit development.

### Up-regulation of *atTOC120* and *atTOC90* by their endogenous leader intron

Leader intron in 5′ UTR is to moderate the level and pattern of gene expression in *Arabidopsis* (Norris et al., 1993;Jeong et al., 2006;Chen and Sun, 2010). The mechanism of intron-mediated enhancement (IME) is largely unknown, but IME might increase transcription efficiency or mRNA stability (Rose et al., 2008). As *atTOC132*, *atTOC120* and *atTOC90* have similar gene structures and include intron in 5′ UTR, we want to examine whether the leader intron sequences play a role in regulating the *atTOC132*, *atTOC120* and *atTOC90* expression in stable transgenic plants. With similar strategies, the pCAMBIA1391Z-based transgenes containing upstream sequence and 5′ UTR prior to endogenous leader intron sequence were transformed into wild type *Arabidopsis* to create 132P, 120P and 90P transgenic plants (Figure 1). The p132P plasmid contained 383-bp promoter and 74-bp 5′ UTR sequences of *atTOC132*. The p120P plasmid contained 673-bp promoter and 23-bp 5′ UTR sequences of *atTOC120*. The p90P plasmid included 575-bp promoter and 56-bp 5′ UTR sequences of *atTOC90*. The precise position of 5′ UTR and intron of *atTOC159* is undetermined, so its intron effect is not able to verify in this study. Twenty three, fifty and eleven independent transgenic lines containing construct p132P, p120P and p90P, respectively, were identified. They were named 132P-1 ~ 23, 120P-1 ~ 50 and 90-1 ~ 11. Three homozygous 132P (132P-1, 132P-13, 132P-19), three homozygous 120P (120P-3, 120P-12, 120P-28) and three homozygous 90P (90P-1, 90P-3, 90P-9) T_3_ transformants were used for further GUS analyses.

**Figure 4:**
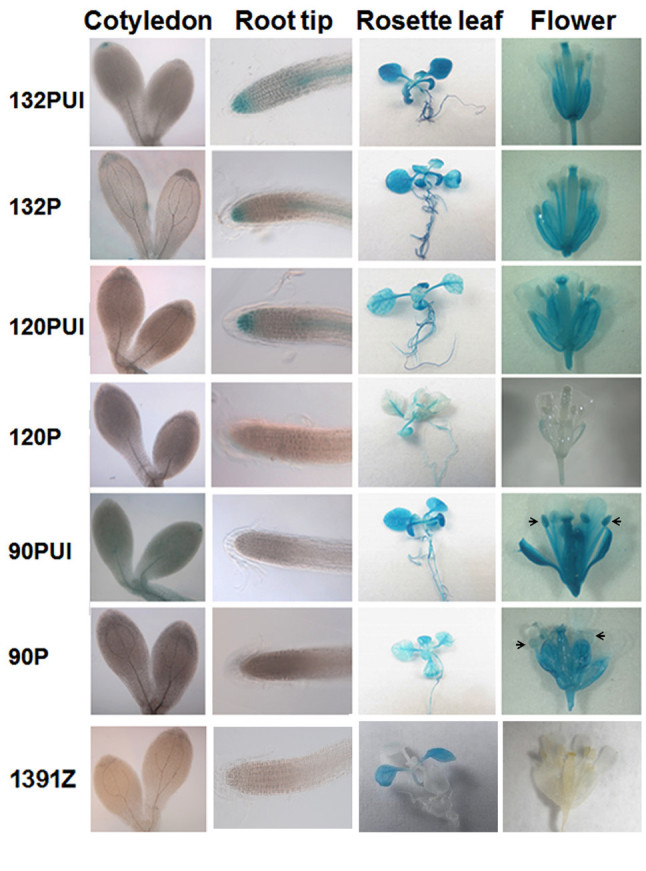
**Induction of** ***atTOC132*** **,** ***atTOC120*** **and** ***atTOC90*** **expression by endogenous intron.** The GUS expression of cotyledons, root tips, leaves and mature flowers was demonstrated by histochemical staining. Cotyledon and root tip were collected from 5-day-old etiolated seedlings transferred to continuous light treatment for 4 hours. Rosette leaf was harvested from 2-week-old plate-grown plants. Mature flower was collected from soil-grown plants. These tissues were incubated in GUS buffer for 1.5 hours (cotyledon and root tip), 3 hours (leaf) and 24 hours (flower) at 37°C. The tissues were observed and photographed by confocal microscope with visible light source (Leica TCS SP2) and EOS 600D software (Canon) (cotyledon and root tip), digital camera (Ricoh CX3, rosette leaf), stereo microscope (Leica M3Z) and digital camera (Nikon Coolpix P5100) (flower). 132PUI, 132P, 120PUI, 120P, 90PUI and 90P plants shown here were their homogenous T_3_ transgenic lines, 132PUI-7, 132P-13, 120PUI-3, 120P-28, 90PUI-3 and 90P-9, respectively. Arrows indicate the anthers.

The GUS activity of three transgenic lines contained the same construct from 5-day-old seedlings, 2-week-old leaves, and mature flowers was examined histochemically. Plant 132PUI-7, 132P-13, 120PUI-3, 120P-28, 90PUI-3 and 90P-9 were representatives in Figure 4. The 132PUI and 132P plants had similar GUS activity in all tissues examined. However, compared to 120P plant, 120PUI plant had obviously higher GUS activity in root tips, rosette leaves, and mature flowers. In comparison with 90P plant, 90PUI had higher GUS activity in cotyledons, rosette leaves and flowers, significantly notable in anthers. These data reveal that the endogenous intron sequence indeed up-regulates *atTOC132* and *atTOC90* expression in a tissue-specific manner, but it has no effect in moderating *atTOC132* expression.

## Discussion

Previous genetic and biochemical studies suggested that atToc159 and atToc90 were important for the accumulation of photosynthetic proteins during chloroplast development. The knockout *Arabidopsis* mutants of *atTOC159* and *atTOC90* were named *ppi2* (plastid protein import 2, or *attoc159*) and *ppi4* (or *attoc90*), respectively. The *ppi2* mutant resulted in an albino seedling that was not able to grow beyond the cotyledon stage on soil (Bauer et al., 2000). The *ppi2* plastids failed to accumulate massive amount of photosynthetic proteins, and therefore lacked thylakoids and starch granules. Nevertheless, the residual amounts of non-photosynthetic proteins in *ppi2* plastid were not less than those of wild type plastid, underlining the importance of atToc159 for chloroplast development (Bauer et al., 2000). The *ppi4* mutant had no obvious phenotype (Hiltbrunner et al. 2004). However, *ppi2*/*ppi4* double mutant appeared a paler phenotype than *ppi2* single mutant, revealing a functional overlap between atToc159 and atToc90 (Hiltbrunner et al. 2004). Nonetheless, overexpression of *atTOC90* partially complemented the *ppi2* defect, demonstrating atToc90 could only contribute to the import route for some atToc159 client proteins (Infanger et al., 2011). The severity of phenotypic defect could be linked to our transgene results. The steady expression level of *atTOC159* in light-grown young leaves is much higher than that of *atTOC90* (Figure 2), suggesting that atToc159 plays a more significant roles as the receptor of photosynthetic proteins in leaves and cotyledons.

By contrast, atToc132 and atToc120 seemed to be more specific for recognizing and importing non-photosynthetic proteins (Ivanova et al., 2004;Kubis et al., 2004). AtToc132 (and atToc120) was selectively binding to the transit peptide of preE1α, prepyruvate dehydrogenase E1a subunit representing a non-photosynthetic protein in plastid, rather than binding to the transit peptide of preSSU, presmall subunit of Rubisco representing the photosynthetic protein in chloroplast (Ivanova et al., 2004). The plastids in root of homozygous *attoc132/attoc120* double mutant had irregular shape and contained large cytoplasmic inclusions (Kubis et al. 2004). Stanga et al. (2009) recently reported that overexpression of *atTOC132* rescued the defect of gravitropism sensing and signaling in *arg1* (*altered response to gravity 1*) and *mar2* (modifier of ARG1, identified as atToc132 now) double mutant, revealing atToc132 to involve in gravity signal transduction within the plastid. These studies clearly suggest that *atTOC132* and *atTOC120* indeed play important roles in regulating root development. From above, atToc132 and atToc120 appear to preferentially affect the import of non-photosynthetic preproteins. However, expression of *atTOC120* failed to complement *attoc132*/*attoc120* double mutant, suggesting atToc120 was not sufficient to recognize most of non-photosynthetic proteins. This is probably due to the low expression yield of *atTOC120* in *Arabidopsis*. Our data in this study indicated that the expression level of *atTOC132* in leaves and roots was higher than that of *atTOC120* (Figure 2), pointing out that atToc132 devotes more in the machinery of non-photosynthetic protein import.

Recent large-scale proteomic studies have raised the question whether it is suitable to categorize atToc159 and atToc132 as major receptors of photosynthetic and non-photosynthetic preproteins, respectively. Based on the comparative analysis of plastid proteome from two *attoc159* knockout mutants to wild type plant, many photosynthetic proteins accumulated but several metabolic proteins decreased in *attoc159* plastids (Bischof et al., 2011). This suggested that atToc159 was responsible for recognition of both photosynthetic and non-photosynthetic proproteins. Therefore, the authors defined the plastid preproteins as atToc159-dependent and atToc159-independent precursor proteins. Furthermore, the comparative analysis of chloroplast proteome between *attoc132* mutant and wild type plant showed that the abundances of four photosynthetic-related and five non-photosynthetic proteins increased 1.5 to 2.5 folds, but five non-photosynthetic proteins decreased approximately 2 folds (Kubis et al., 2004). The accumulation of most chloroplastic proteins remained the similar yield. Theses research work might underestimate the function of atToc90 and atToc120. Except for the pale phenotype of *ppi2* (*attoc159* mutant), *attoc132*, *attoc120* and *ppi4* (*attoc90*) single mutant did not have any visible phenotype. However, *ppi2*/*ppi4* double mutant appeared a paler phenotype than *ppi2* single mutant (Hiltbrunner et al. 2004), and *attoc132*/*attoc120* double mutant showed pale green (Kubis et al., 2004) or were even embryo lethal (Ivanova et al., 2004), revealing a functional overlap between atToc159 and atToc90, or between atToc132 and atToc120. It might be more informative to characterize substrate specificity of these atToc159 isoforms by the plastid proteome from double mutant to single mutant or wild type plants.

Even though *atTOC159* gene members tend to differentially express in various vegetative tissues, they all participate in flower and fruit development (Figure 3). Our data suggested that these genes had high expression in flower and early fruit development, and significantly higher expression level in later fruit development (Figure 3). These results were supported by previous genetic studies. For example, the single mutant of each gene is able to produce seeds with normal viability. However, homozygous *attoc159/attoc132* and *attoc132/attoc120* double mutants cause embryo lethality (Ivanova et al., 2004;and Kubis et al., 2004). Therefore, we conclude that all atToc159 isoforms are not only contributed to the import of photosynthetic/non-photosynthetic related proteins during vegetative growth, but also modulate reproductive growth.

The enhancing effects of gene expression by introns were first reported by (Mascarenhas et al. 1990) and the function was defined as IME. Even though the mechanism of IME remains largely unknown, the effect of IME has been broadly observed in eukaryotes, including animals and plants (Jonsson et al., 1990;Palmiter et al., 1991;Rethmeier et al., 1998;Morello et al., 2002;Jeong et al., 2006;Chen and Sun, 2010). Our stable transformation assay suggested the intron sequence of *atTOC132* did not alter its expression yield in cotyledons, root tips, rosette leaves, and flowers (132PUI and 132P in Figure 4). Nevertheless, the intron sequence of *atTOC120* and *atTOC90* indeed increased the expression yield obviously. The endogenous intron sequence up-regulated *atTOC120* expression level specifically in root tips and flowers, but not in cotyledons (120PUI and 120P in Figure 4). In contrast with *atTOC120*, the intron sequence induced *atTOC90* expression level specifically in cotyledons, leaves and anthers, but not in root tips and rest of flower tissues (90PUI and 90P in Figure 4). These findings imply that the intron effect of *atTOC120* is more effective in roots and whole flowers, and *atTOC90* is in cotyledons, leaves and anthers. We thus hypothesized that these introns might contain tissue-specific enhancers. Additional experiments are clearly required to prove this hypothesis.

The level of GUS activity in 1-or 2-week-old leaves and 3-week-old roots of 120PUI plant was similar to 1391Z control plant (Figure 2). This seemed to imply that 120PUI did not express *GUS* gene. However, 120PUI plant showed a moderate GUS expression level in the 5-day-old root tips and 2-week-old rosette leaves (Figure 4). These conflict results might be due to the binary vector pCAMBIA1391Z used in this transgenic study. Even though pCAMBIA1391Z contains a promoter-less *GUS* gene, a nearby cauliflower mosaic virus (CaMV) 35S promoter which drives the expression of hygomycin phosphotransferase II (*HptII*) gene, could also drive the expression of *GUS* gene. In consequence, the transgenic plants containing pCAMBIA1391Z show a low to moderate level of GUS expression in various tissues (http://www.cambia.org). Based on this rationale, 120PUI plant indeed expresses the *GUS* gene in leaves and root tips, and the expression level is similar to 1391Z control plant. Besides, the GUS activity of leaves in 120P plant was lower than 1391Z plant (Figure 4). It is possible that an actively expressed gene can suppress the expression of a close-by gene, especially a weakly expressed gene. For example, unlike most of polyubiquitin (*UBQ*) genes in Arabidopsis which had an abundantly expressed yield, the expression level of *UBQ4* was barely detected in all tissues (Sun and Callis, 1997). Carter et al. (1998) indicated thereafter that *UBQ4* expression was suppressed by a strongly expressed nearby gene, germin-like protein 3b (*GLP3b*), which was approximately 500 bp upstream of *UBQ4* transcription start site. Altogether, we conclude that pCAMBIA1391Z vector may not be appropriate to determine the absolute expression level of weakly expressed genes, but still feasible to compare the relative expression level of these genes in transgenic plants.

## Conclusions

Due to their recognition of different substrate proteins during protein import into plastids, the closely related *atTOC159* homologous genes are independently or coordinatedly regulated in response to developmental signals to ensure the proper import of plastid proteins. Based on the results from stable transgenic plants expressing GUS reporter, we conclude that the upstream regulatory sequences of *atTOC159* homologous genes govern the differential expression of these genes among vegetative and reproductive tissues. Furthermore, the endogenous leader intron sequences of *atTOC120* and *atTOC90* up-regulate gene expression in a tissue-specific manner.

## Authors’ original submitted files for images

Below are the links to the authors’ original submitted files for images. Authors’ original file for figure 1 Authors’ original file for figure 2 Authors’ original file for figure 3 Authors’ original file for figure 4

## Abbreviations

GUS: β-glucuronidase
PCR: Polymerase chain reaction
Tic/Toc: Translocon at the inner/outer envelope membrane of the chloroplast
5′ UTR: 5′ untranslated region.
