# Supplementary figures and images for: Characterization of differential expression and leader intron function of Arabidopsis atTOC159 homologous genes by transgenic plants
Source: Bot Stud. 2013 Sep 25;54:40. doi: 10.1186/1999-3110-54-40 (PMC5430346; doi:10.1186/1999-3110-54-40)

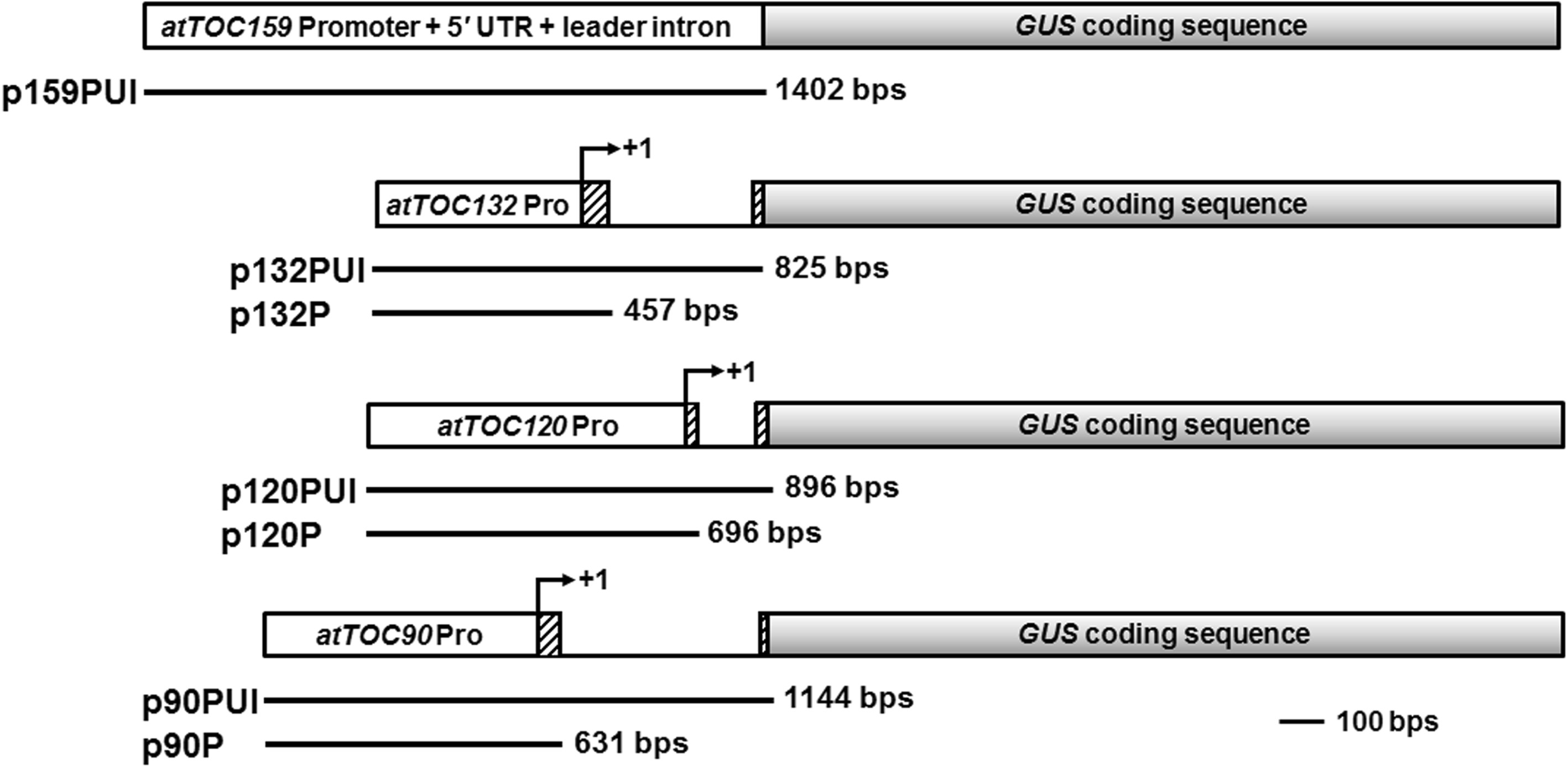

Supplement: Supplementary file 1 — Authors’ original file for figure 1 [file 40529_2013_38_MOESM1_ESM.tif]

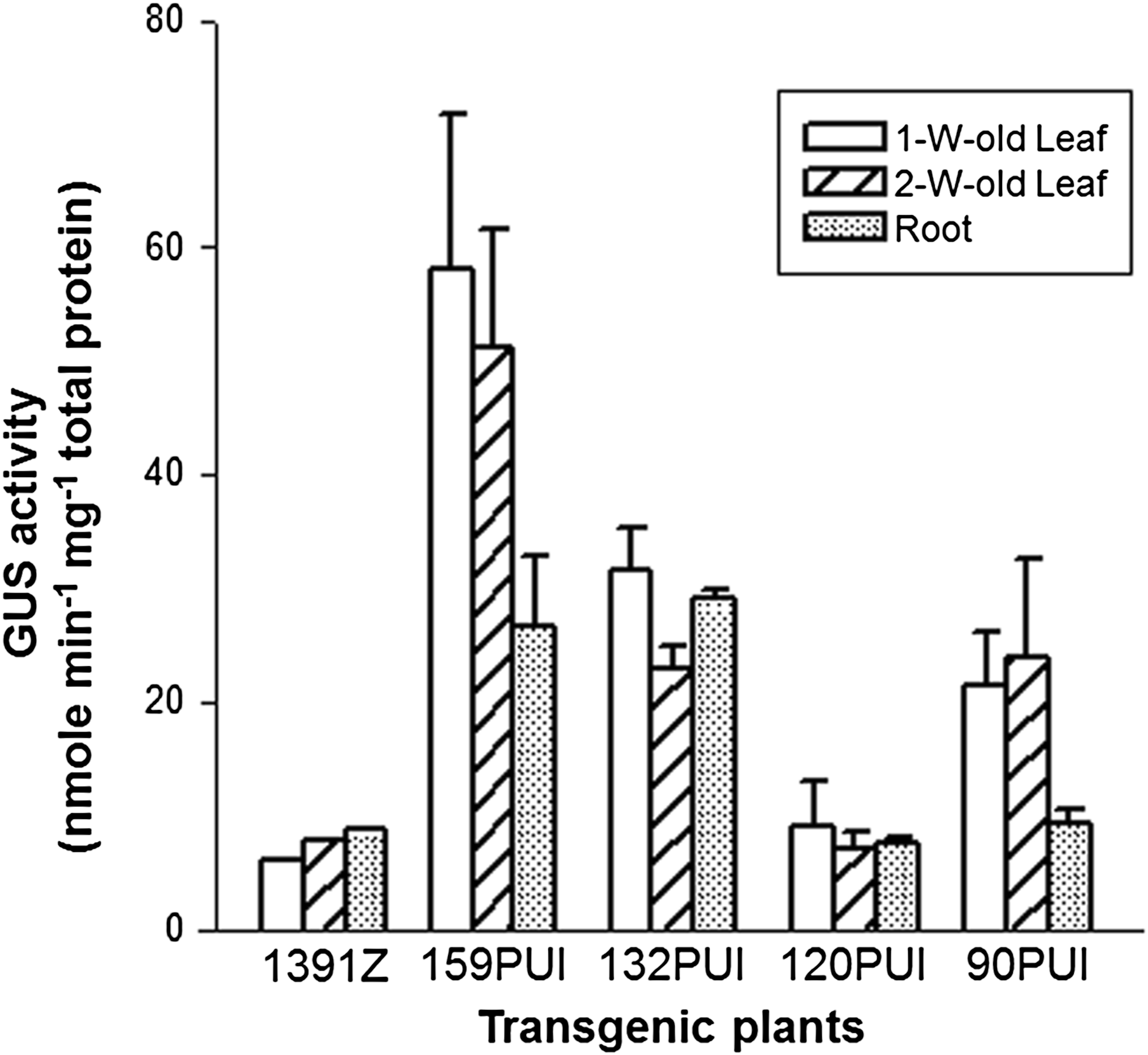

Supplement: Supplementary file 2 — Authors’ original file for figure 2 [file 40529_2013_38_MOESM2_ESM.tif]

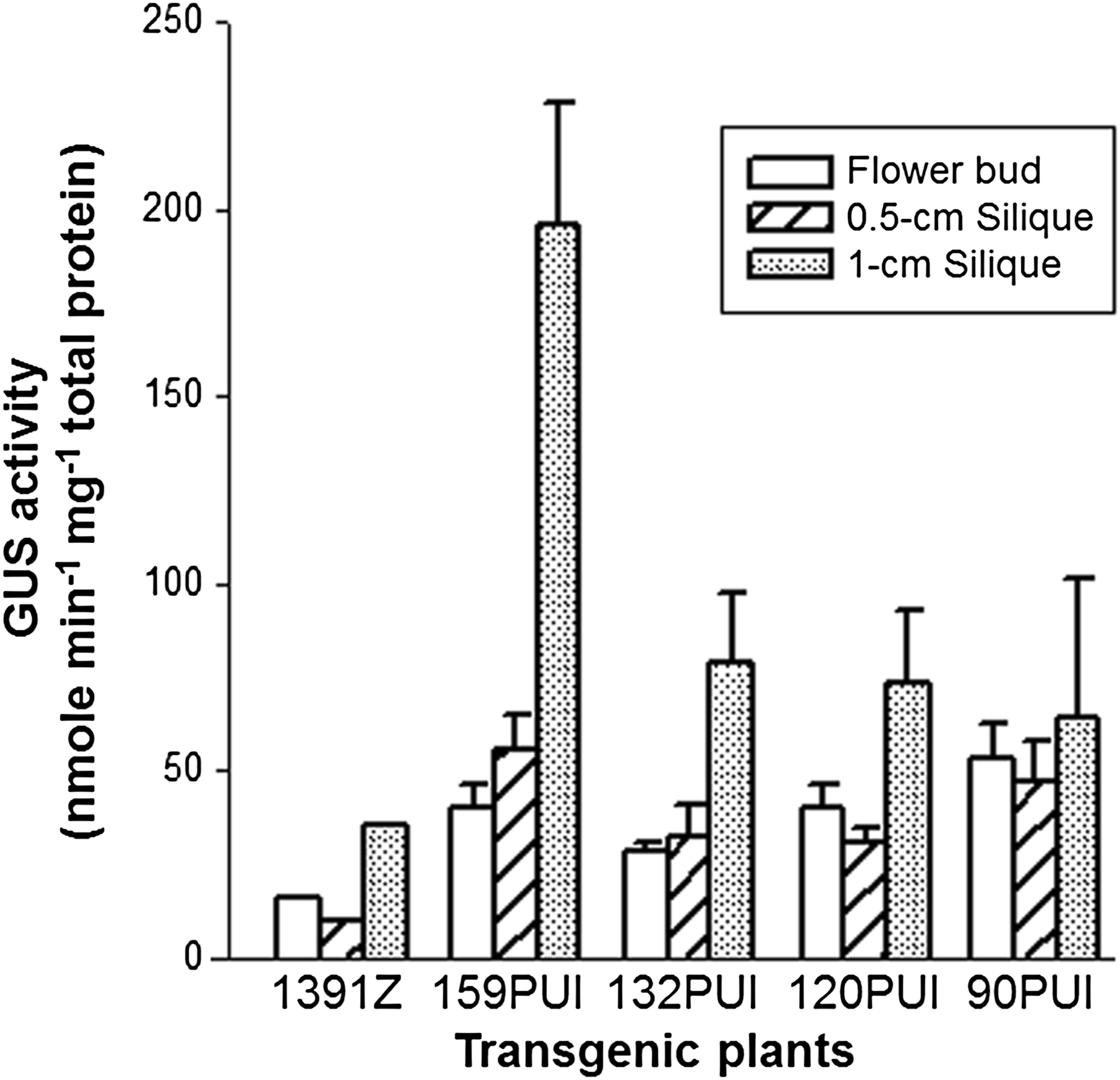

Supplement: Supplementary file 3 — Authors’ original file for figure 3 [file 40529_2013_38_MOESM3_ESM.tif]

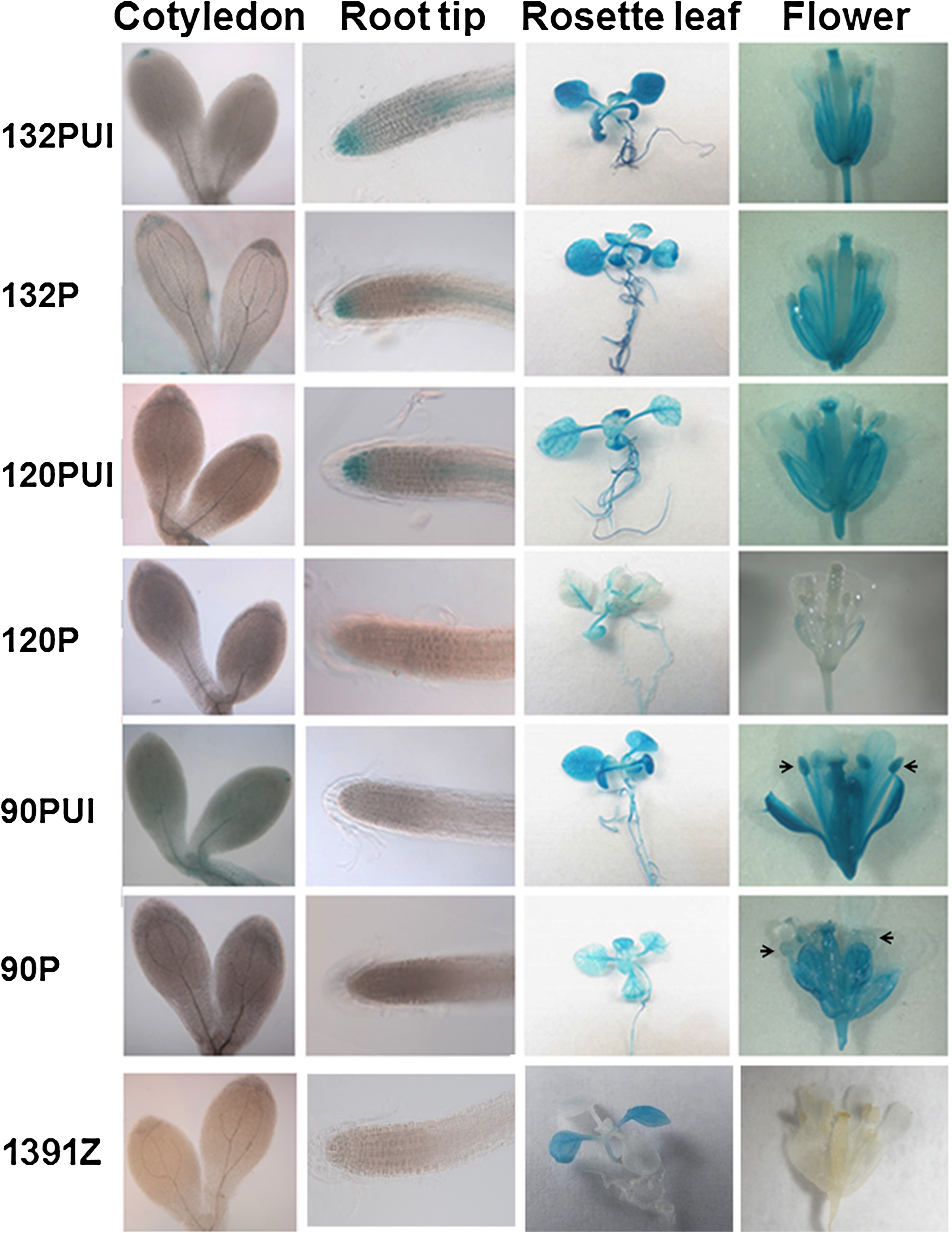

Supplement: Supplementary file 4 — Authors’ original file for figure 4 [file 40529_2013_38_MOESM4_ESM.tif]
